# Assessing the impact of sodium intake on kidney function deterioration and proteinuria in the general population: A prospective cohort study

**DOI:** 10.1371/journal.pone.0330342

**Published:** 2025-08-29

**Authors:** Chan Young Park, Jong Hoon Seok, Seung Yoon Lee, Ji Eun Kim

**Affiliations:** 1 Korea University College of Medicine, Seoul, Republic of Korea; 2 Department of Internal Medicine, Korea University Guro Hospital, Seoul, Republic of Korea; Federal Fluminense University: Universidade Federal Fluminense, BRAZIL

## Abstract

Excessive sodium intake is recognized as a potential risk factor for various diseases, including kidney disease. However, limited research has been conducted on the relationship between sodium intake and kidney disease in the general population. This study aimed to explore the association between sodium intake and the risk of kidney disease, utilizing multivariable logistic regression and spline interpolation models. Twelve-year prospective cohort data were analyzed, and participants were categorized based on their sodium intake. Kidney disease was defined by the presence of proteinuria and a reduction in estimated glomerular filtration rate below 60 ml/min/1.73m². In the overall population, sodium intake exceeding 2g/day was not significantly associated with kidney dysfunction (adjusted OR: 0.84, 95% CI: 0.67–1.04, p = 0.113). However, in subgroup analyses, individuals with diabetes who consumed more than 5g/day of sodium had a significantly increased risk of kidney dysfunction (adjusted OR: 3.76, 95% CI: 1.36–10.30, p = 0.01). Across both the general population and subgroup analyses, sodium intake was not significantly associated with proteinuria. These findings suggest that while sodium intake may not have a substantial impact on kidney function in the general population, it may play a critical role in accelerating kidney dysfunction in individuals with comorbidities such as diabetes.

## Introduction

Sodium is essential for human health, but both deficiency and excess can lead to significant health issues. Excessive sodium intake (>2g/day, as recommended by the WHO) has been shown to significantly increase blood pressure, a major risk factor for kidney disease [1]. Kidney disease is a growing public health concern worldwide, and studies have demonstrated that high sodium intake exacerbates kidney damage, particularly in patients with chronic kidney disease (CKD) [2]. Experimentally, a high-salt diet has been shown to worsen cardiovascular and renal damage in hypertension models [3], while clinically, hypertension is a critical factor in the progression of renal dysfunction in patients with kidney disease [4,5]. In this context, extensive research has been conducted to understand the relationship between sodium consumption and renal function decline, particularly in patients with advanced stages of CKD. Most studies have concluded that excessive sodium intake aggravates CKD [6–7].

While many studies have focused on the effects of sodium intake in patients already diagnosed with CKD, less is known about its impact on kidney function in the general population without pre-existing kidney disease [8]. Given the rise in global sodium intake over the past three decades, understanding its potential role in the early development of kidney dysfunction is critical.

This study aims to investigate the relationship between sodium intake and the risk of kidney function deterioration in the general population. Additionally, considering that individuals with underlying conditions such as hypertension and diabetes are at high risk for kidney dysfunction, this study will assess the differential impact of sodium intake on kidney function in these specific subgroups.

## Materials and methods

### Ethical considerations

This study used anonymized data from the Korean Genome and Epidemiology Study (KoGES), accessed on March 17, 2023. The study received an exemption determination from the Institutional Review Board of Korea University Guro Hospital (Approval Number: 2020GR0151, Date: April 09, 2020) as it involved the secondary analysis of anonymized data. No authors had access to information that could identify individual participants during or after data collection. This research complies with institutional and national ethical guidelines, as well as the Declaration of Helsinki.

### Study setting and data source

The basic data are based on the Ansan and Ansung community cohort studies of the Korean Genome and Epidemiology Study (KoGES) conducted by the Korea Centers for Disease Control and Prevention in 2001–2002. We then collected data from biennial follow‐up examinations conducted until 2011–2012. We excluded those with proteinuria, those whose eGFR rate was below 60 ml/min/1.73m^2^, and those whose data in food frequency questionnaire were missing. Initially, participants were categorized into two groups: the Regular Sodium Intake Group, consisting of individuals who consumed more than 2g of sodium per day, and the Low Sodium Intake Group, consisting of those who consumed 2g or less, in accordance with the World Health Organization’s recommendation. Following this, we examined the association between sodium intake and kidney function using spline curves. For the spline curve analysis, sodium intake levels were further stratified, and high sodium intake was defined as consuming more than 5g of sodium per day.

### Data collection

Various variables obtained from the KoGES data were included in the analysis, such as age, gender, BMI, smoking status, alcohol consumption, the onset of hypertension, diabetes mellitus, coronary artery disease, kidney disease, eGFR, albumin, hemoglobin levels, and the results of urine dipstick testing. Due to substantial missing values, 24-hour urine collection and urine protein-to-creatinine ratio data were not used. Additionally, daily intake of calorie, protein, potassium, and sodium were calculated based on the food frequency questionnaire.

Dietary intake was assessed using a semi-quantitative food frequency questionnaire (SQFFQ) developed specifically for the Korean Genome and Epidemiology Study (KoGES). The SQFFQ consisted of 106 food and beverage items, selected based on their contribution to the intake and variability of 17 major nutrients, as identified from the Korea National Health and Nutrition Examination Survey (KHANES). Participants were asked to report their average frequency of consumption and portion size for each item over the past year. Intake frequency was categorized into nine levels, ranging from “almost never” to “three times per day.”

Daily food intake amounts were calculated by integrating frequency, portion size, and seasonality data. The calculation process involved several steps. First, the reported intake frequency for each food item was standardized to a daily frequency. The portion size for each food was determined using standardized portion size reference data and converted into grams. The daily intake amount for each food item was calculated by multiplying the daily intake frequency by the corresponding portion size. Subsequently, the nutrient content for each food item was derived by combining the intake amount (in grams) with nutrient composition data obtained from a standardized food composition database, which provides nutrient values per 100 grams of food. Individual daily nutrient intakes were obtained by summing the nutrient intakes across all food items.

Kidney dysfunction was defined as a decrease in estimated glomerular filtration rate (eGFR) to less than 60 ml/min/1.73m², and proteinuria was defined as the result of 1+ or higher in the urine dipstick test. eGFR was calculated using the CKD-EPI equation, as recommended by the National Kidney Foundation (NKF) and the American Society of Nephrology (ASN) [5].

### Study outcomes

The primary outcome was kidney dysfunction, and the secondary outcome was the occurrence of proteinuria. Subgroup analyses were performed to evaluate the differential impact of sodium intake on kidney function in individuals with diabetes and hypertension.

### Statistical analysis

The baseline characteristics of the study participants are described using the mean with standard deviation (SD) for continuous variables, and frequency is described using counts and percentages for continuous variables. We used t-test for comparisons of continuous variables and the χ^2^ test for categorical variables as appropriate. Univariable and multivariate logistic regression models were used to evaluate the association between sodium intake and the occurrence of kidney dysfunction and proteinuria. In the multivariate models, adjustments were made for age, gender, BMI, smoking status, alcohol consumption, hypertension, diabetes, coronary artery disease, underlying kidney disease, baseline eGFR, daily protein intake, albumin, and hemoglobin levels. Cubic spline curves were applied to illustrate the relationship between sodium intake and the odds of kidney dysfunction and proteinuria. Statistical significance was defined as a two-sided p-value of less than 0.05. All analyses were conducted using R version 4.3.2.

## Results

### Baseline characteristics

**Fig 1:**
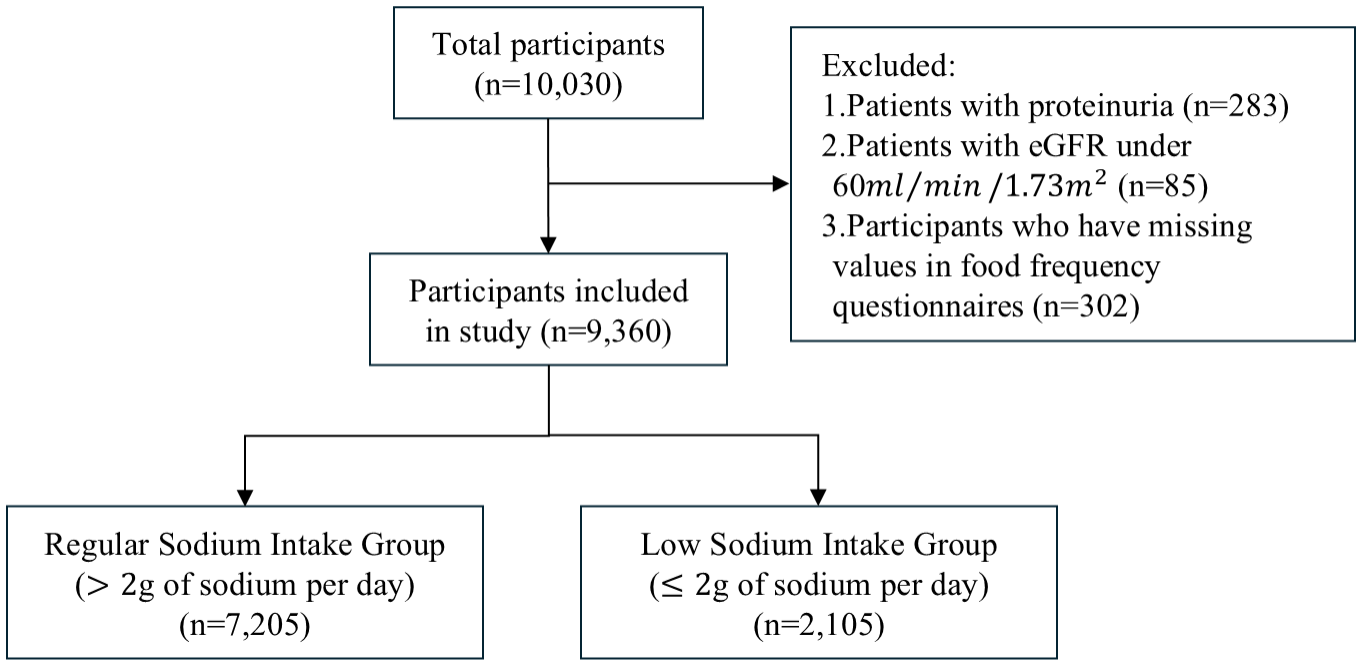
Flow diagram for inclusion and exclusion of studies. This flow diagram depicts the numbers and reasons for excluding studies based on our pre-determined criteria. The figure also illustrates the numbers of subjects who consume more than 2g of sodium per day (Regular Sodium Intake Group) and who consume 2g or less of sodium per day (Low Sodium Intake Group).

A total of 10,030 men and women, aged 40–69 years, participated in the 2001–2002 Ansan and Ansung community cohort studies of the KoGES. After excluding subjects based on the pre-determined criteria, data from 9,360 people were involved in the analysis. The Regular Sodium Intake Group includes 7,205 individuals and the Low Sodium Intake Group includes 2,105 individuals. Study selection process was shown on Fig 1. The mean (SD) age was 51.8 (8.8) years in Regular Sodium Intake Group and 53.2 (9.0) years in Low Sodium Intake Group. 49.7% of Regular Sodium Intake Group were female and 62.9% of Low Sodium Intake Group were female. There were no significant differences in smoking status, alcohol status, diagnosis of hypertension, diagnosis of diabetes mellitus, diagnosis of coronary artery disease, diagnosis of kidney disease, estimated glomerular filtration rate, blood albumin level and hemoglobin level between two groups. The general data of the two groups are summarized in Table 1.

**Table 1:** Baseline characteristics of total participants (n = 9360).

Characteristics	Total (n = 9360)	Regular sodium intake (n = 7205)	Low sodium intake (n = 2155)	*P*-value
Age, years	52.1 ± 8.9	51.8 ± 8.8	53.2 ± 9.0	<0.001
Gender, female	4937 (52.7%)	3581 (49.7%)	1356 (62.9%)	<0.001
BMI, kg/$m^{2}$	24.6 ± 3.1	24.6 ± 3.1	24.3 ± 3.1	<0.001
Smoking status				<0.001
Never	5471 (59.0%)	4055 (56.8%)	1416 (16.7%)	
Before	1442 (15.5%)	1190 (16.7%)	252 (11.8%)	
Current	2363 (25.5%)	1894 (26.5%)	469 (21.9%)	
Alcohol consumption				
Never	4324 (46.4%)	3163 (44.1%)	1161 (54.1%)	<0.001
Before	600 (6.4%)	454 (6.3%)	146 (6.8%)	
Current	4399 (47.2%)	3558 (49.6%)	841 (39.2%)	
Hypertension	1374 (14.7%)	1044 (14.5%)	330 (15.3%)	0.361
Diabetes mellitus	600 (6.4%)	464 (6.4%)	136 (6.3%)	0.870
Coronary artery disease	73 (0.8%)	46 (0.6%)	27 (1.3%)	0.007
Underlying kidney disease	255 (2.7%)	186 (2.6%)	69 (3.2%)	0.140
Daily intake amount from food frequency questionnaires				
Energy, kcal	1958.4 ± 15.8	2085.1 ± 732.5	1534.8 ± 445.3	<0.001
Protein, g	66.6 ± 30.4	72.6 ± 31.3	46.8 ± 15.2	<0.001
Potassium, mg	2548.4 ± 1224.3	2841.3 ± 1214.4	1569.0 ± 577.1	<0.001
Sodium, mg	3197.4 ± 1664.9	3722.6 ± 1534.5	1441.4 ± 400.9	<0.001
Laboratory tests				
eGFR, ml/min/1.73m^2^	97.2 ± 14.2	97.3 ± 14.1	97.2 ± 14.5	0.924
Albumin, g/dL	4.2 ± 0.3	4.3 ± 0.3	4.2 ± 0.3	<0.001
Hemoglobin, g/dL	13.6 ± 1.6	13.7 ± 1.6	13.3 ± 1.6	<0.001

Abbreviations: BMI, body mass index; eGFR, estimated glomerular filtration rates

### Impact of low vs. regular sodium intake on kidney function

**Fig 2:**
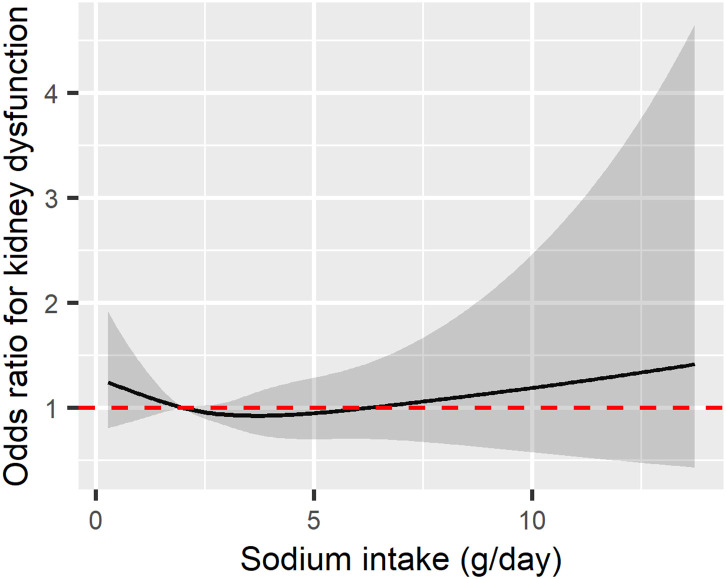
Spline curve for the odds of kidney dysfunction in relation to sodium intake. The odds ratio is adjusted for age, sex, body mass index (BMI), smoking status, alcohol consumption, hypertension, diabetes, coronary artery disease, pre-existing kidney disease, baseline estimated glomerular filtration rate (eGFR), daily protein intake, blood albumin level, and hemoglobin. The black solid line represents the odds ratio (OR), while the gray transparent area indicates the 95% confidence interval (CI). The red dotted line denotes the reference point where OR = 1.

Over the 12-year follow-up period, a total of 680 individuals developed kidney dysfunction. In the unadjusted logistic regression analysis, the OR for the development of kidney dysfunction in the regular sodium intake group, compared to the low sodium intake group, was 0.71 (95% CI: 0.6–0.84; p < 0.001). However, after adjusting for age, sex, BMI, smoking status, alcohol consumption, hypertension, diabetes, coronary artery disease, baseline kidney disease, baseline eGFR, daily protein intake, blood albumin and hemoglobin levels, the OR for the regular sodium intake group changed to 0.84 (95% CI 0.67–1.04), losing statistical significance (Table 2). Therefore, among the total participants, regular sodium intake was not associated with an increased risk of kidney dysfunction compared to the low sodium intake. The spline curve illustrating the relationship between sodium intake levels and the odds of kidney dysfunction in the total group is presented in Fig 2.

**Table 2:** The multivariate logistic regression analysis for kidney dysfunction.

Variables	Odds Ratio (95% CI)	*P*-value
Regular Sodium Intake (Na > 2g)	0.84 (0.67–1.04)	0.113
Age, years	1.09 (1.08–1.1)	<0.001
Gender, female	1.92 (1.4–2.64)	<0.001
BMI	1.05 (1.02–1.08)	<0.001
Smoking status	0.9 (0.81–1)	0.051
Alcohol consumption	1.02 (0.91–1.14)	0.732
Hypertension	1.65 (1.35–2.01)	<0.001
Diabetes mellitus	2.36 (1.82–3.04)	<0.001
Coronary artery disease	0.6 (0.25–1.29)	0.22
Underlying kidney disease	1.89 (1.23–2.84)	0.003
Baseline eGFR	0.94 (0.94–0.95)	<0.001
Daily protein intake	1 (0.99–1.01)	0.625
Albumin	0.5 (0.36–0.68)	<0.001
Hemoglobin	1 (0.92–1.09)	0.967

Abbreviations: BMI, body mass index; eGFR, estimated glomerular filtration rates

### Differential impact of sodium intake on kidney function in diabetes and hypertension subgroups

After adjusting for variables in the entire participant group, no significant association was observed between the risk of kidney dysfunction and low sodium intake. However, since an initial association was observed prior to adjustment, we conducted subgroup analyses to explore potential variations in risk among specific populations.

**Fig 3:**
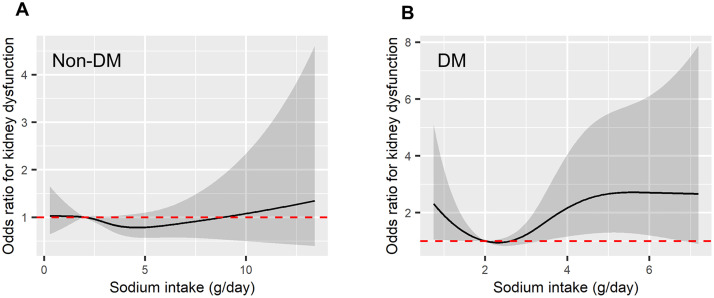
Spline curves showing the odds of kidney dysfunction relative to sodium intake, differentiated by the presence of diabetes mellitus. Fig 3A presents individuals without diabetes, and Fig 3B includes individuals with diabetes. In both, the odds ratios are adjusted for age, sex, BMI, smoking status, alcohol consumption, hypertension, coronary artery disease, pre-existing kidney disease, baseline eGFR, daily protein intake, blood albumin, and hemoglobin levels. The black solid lines represent the odds ratios (ORs), the gray transparent areas denote the 95% confidence intervals (CIs), and the red dotted lines indicate the reference point where OR = 1.

**Fig 4:**
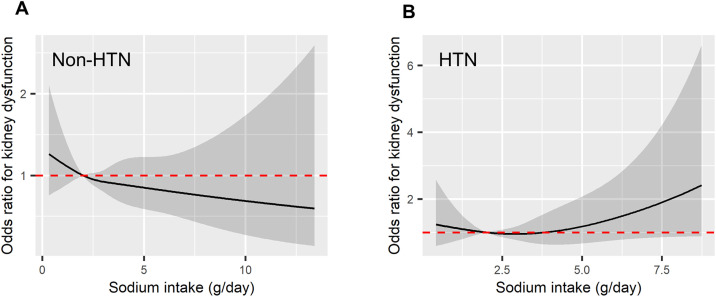
Spline curves showing the odds of kidney dysfunction relative to sodium intake, differentiated by the presence of hypertension. Fig 4A presents individuals without hypertension, and Fig 4B includes individuals with hypertension. In both, the odds ratios are adjusted for age, sex, BMI, smoking status, alcohol consumption, diabetes, coronary artery disease, pre-existing kidney disease, baseline eGFR, daily protein intake, blood albumin, and hemoglobin levels. The black solid lines represent the odds ratios (ORs), the gray transparent areas denote the 95% confidence intervals (CIs), and the red dotted lines indicate the reference point where OR = 1.

In the subgroup analysis for diabetes, the non-diabetes group showed a relationship between sodium intake and the odds of kidney dysfunction that was similar to that observed in the total group. However, in the diabetes group, the odds of kidney dysfunction increased with sodium intake, and the relationship between sodium intake and kidney dysfunction exhibited a U-shaped association (Fig 3B). Similarly, while the non-hypertension group exhibited an association comparable to that of the total group, the hypertension group showed a similar pattern of association as the diabetes group, with increasing sodium intake linked to higher odds of kidney dysfunction (Fig 4B). Through these spline curve analyses, we compared the OR of consuming more than 5g of sodium per day (‘high sodium intake’) for kidney dysfunction consuming 2g-3g of sodium per day. In the total group, consuming high sodium was not associated with the development of kidney dysfunction (1.24; 95% CI: 0.86–1.77; p = 0.254). However, in the diabetes group, high sodium intake was associated with a 3.76 times higher risk of kidney dysfunction (95% CI: 1.36–10.30; p = 0.01). In the hypertension group, the risk of kidney dysfunction slightly increased to 1.71 times with high sodium intake, but this was not statistically significant (OR 1.71; 95% CI: 0.87–3.35; p = 0.118).

### Association of sodium intake with proteinuria

**Fig 5:**
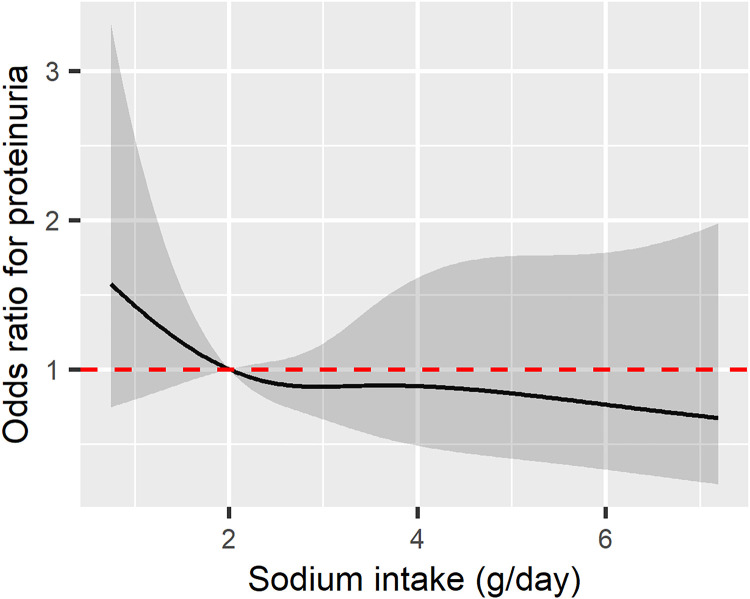
Spline curve for the odds of proteinuria in relation to sodium intake. The odds ratio is adjusted for age, sex, body mass index (BMI), smoking status, alcohol consumption, hypertension, diabetes, coronary artery disease, pre-existing kidney disease, baseline estimated glomerular filtration rate (eGFR), daily protein intake, blood albumin level, and hemoglobin. The black solid line represents the odds ratio (OR), while the gray transparent area indicates the 95% confidence interval (CI). The red dotted line denotes the reference point where OR = 1.

Over a 12-year period, a total of 91 individuals newly developed proteinuria. In both unadjusted and adjusted logistic regression analyses, regular sodium intake was not associated with an increased risk of proteinuria compared to low sodium intake (Unadjusted OR = 0.8; 95% CI: 0.53–1.36; p = 0.448, Adjusted OR = 0.73; 95% CI: 0.43–1.28; p = 0.260) (Table 3). The spline curve depicting the relationship between sodium intake levels and the odds of proteinuria in the total group is presented in Fig 5.

**Table 3:** The multivariate logistic regression analysis for proteinuria.

Variables	Odds Ratio (95% CI)	*P*-value
Regular Sodium Intake (Na > 2g)	0.73 (0.43–1.28)	0.260
Age, years	1.01 (0.98–1.03)	0.678
Gender, female	0.66 (0.32–1.38)	0.268
BMI	0.98 (0.91–1.05)	0.565
Smoking status	0.99 (0.79–1.25)	0.955
Alcohol consumption	0.99 (0.76–1.29)	0.935
Hypertension	1.13 (0.65–1.91)	0.649
Diabetes mellitus	6.34 (3.86–10.18)	<0.001
Coronary artery disease	4.1 (0.95–12.05)	0.024
Underlying kidney disease	1.06 (0.26–2.94)	0.919
Baseline eGFR	0.99 (0.97–1.01)	0.206
Daily protein intake	1 (0.98–1.01)	0.738
Albumin	0.78 (0.36–1.66)	0.517
Hemoglobin	1.01 (0.84–1.23)	0.899

Abbreviations: BMI, body mass index; eGFR, estimated glomerular filtration rates

Subgroup analyses by diabetes and hypertension status were also conducted to assess the impact of sodium intake on proteinuria risk. An increasing trend in proteinuria with higher sodium intake was observed in individuals with diabetes, although this did not reach statistical significance (S1A and S1B Figs). No significant association between sodium intake and proteinuria was observed in the hypertension subgroup (S2A and S2B Figs).

## Discussion

In this study, we examined the association between sodium intake and the incidence of renal function decline and proteinuria over 12 years among a general population presumed not to have kidney disease. Across the entire population, sodium intake did not have a significant effect on either renal function decline or proteinuria. However, in patients with diabetes, a high level of sodium intake (>5g/day) was found to increase the risk of kidney dysfunction. A similar pattern of increased risk was observed in patients with hypertension, though it was not statistically significant. The occurrence of proteinuria was not associated with sodium intake across the entire patient cohort, nor in the diabetes and hypertension subgroups.

This study provides evidence that may call into question the current public health policies aimed at reducing sodium intake to less than 2g. The lowest odds ratio for renal function decline in the entire population was not observed at the recommended sodium intake of less than 2g, but rather closer to a relatively high-salt diet of approximately 5g. Additionally, a smooth U-shape curve was observed, similar to the shape suggested by previous studies between sodium intake and CVD risk as well as between sodium intake and mortality risk [9,10]. Even among disease groups presumed to benefit from sodium intake restrictions, ambiguous results were observed for the incidence of renal function decline in hypertensive patients, and only in patients with diabetes was an increased risk of renal dysfunction in the High sodium intake group (>5g/day) definitively confirmed. This suggests that the beneficial effects of the current low-sodium dietary recommendations may be limited to a very specific disease group, at least in terms of renal disease outcomes.

Generally, excessive sodium intake is known to negatively impact renal function through various pathophysiological mechanisms. Increases in sodium intake have been reported to cause blood pressure impairment, increase oxidative stress and inflammation, and induce endothelial dysfunction and arterial stiffness [11–13]. Additionally, when sodium intake is reduced, the response of blood pressure and albuminuria to renin-angiotensin system inhibitors is enhanced [14,15]. Based on these findings, various studies have been conducted on the relationship between sodium intake and kidney function progression, particularly in patients with CKD, where high blood pressure and proteinuria can significantly worsen the disease’s progression. A Korean study using the KNOW-CKD cohort found a linear relationship between 24-hour sodium excretion and composite renal outcomes in patients with stages 3–5 CKD [7]. However, a study by Smyth et al., which included patients with various stages of CKD and an average eGFR of 68.4 ml/min/1.73m2, did not find a significant association between 24-hour sodium excretion and renal outcomes [16]. The effects of sodium intake on CKD progression have shown inconsistent results across studies, and the ongoing debate over the validity of reducing salt intake to delay CKD progression lacks large-scale randomized controlled trials.

There is also a lack of clear evidence regarding sodium intake in diabetic patients. A study of 6,213 patients with type 2 DM did not find an association between dietary sodium and CKD incidence [17], and a single-center retrospective study from Japan also found no significant association with worsening renal disease or mortality [18]. Conversely, a prospective cohort study of type 1 DM patients found that lower urinary sodium excretion was associated with a higher risk of ESRD [19]. Our study included relatively early-stage diabetic patients without proteinuria, diagnosed with diabetes, suggesting that diabetes prior to renal function decline can induce glomerular hyperfiltration, and an increase in sodium intake could exacerbate this hyperfiltration [20], leading to faster renal function decline. Additionally, high sodium intake has been reported to affect insulin resistance [21,22], potentially exacerbating kidney damage in diabetic patients due to difficulties in blood sugar control. High sodium intake may also induce glomerular hypertension, increasing albuminuria and accelerating renal function decline, which, although not statistically significant, was suggested by the trend observed in diabetic patients in our study.

Our study utilized a prospective Korean cohort to track renal function and proteinuria occurrence over a relatively long period, demonstrating a nonlinear U-shaped relationship through spline curves between different sodium intake levels and public health policy implications. However, it also has limitations. First, data from 24-hour urine sodium or the urine protein-to-creatinine ratio, which are considered more accurate measures, were not available for use. As a result, estimates of sodium intake and proteinuria may be prone to some degree of measurement error. Also, the participants’ sodium intake was measured only once at baseline, and continuous tracking of sodium intake during the study period was not conducted, making it difficult to assess the impact of dietary changes on the outcomes. Additionally, although various factors were controlled for in the multivariate analysis, potential confounding variables—such as the age at onset of comorbidities, their duration, and the presence of other conditions not included in the analysis—may have been excluded. Lastly, this study provides a statistical perspective only, without addressing the genetic factors or metabolic effects by which sodium affects the kidneys.

Based on our findings, we highlight the importance of sodium intake management in patients with diabetes, suggesting that specific sodium management strategies targeted at certain population groups should be developed to improve public health policies. The justification for a uniform daily sodium intake restriction across the general population remains questionable without further evidence from additional research. Future studies should include long-term sodium intake monitoring in prospective research to define causal relationships and explore the pathophysiological mechanisms by which sodium intake affects renal function in specific groups more clearly. Also, future studies could complement the findings of the present research by including participants outside the 40–69 age range, incorporating individuals of non-Korean ethnic backgrounds, or conducting subgroup analyses based on more detailed assessments of baseline kidney function.

## Supporting information

S1 Fig The spine curve illustrating the odds of proteinuria in relation to sodium intake, stratified by diabetes mellitus status. S1A and S1B Figs show individuals without and with diabetes mellitus, respectively. Odds ratio (ORs) are adjusted for age, sex, body mass index (BMI), smoking status, alcohol consumption, hypertension, diabetes, coronary artery disease, pre-existing kidney disease, baseline estimated glomerular filtration rate (eGFR), blood albumin level, and hemoglobin. The black solid line represents the OR, while the gray shaded area indicates the 95% confidence interval (CI). The red dotted line denotes the reference point where OR = 1.
(PDF)

S2 Fig The spline curve illustrating the odds of proteinuria in relation to sodium intake, stratified by hypertension status. S2A and S2B figures show individuals without and with hypertension, respectively. Odds ratio (ORs) are adjusted for age, sex, body mass index (BMI), smoking status, alcohol consumption, hypertension, diabetes, coronary artery disease, pre-existing kidney disease, baseline estimated glomerular filtration rate (eGFR), blood albumin level, and hemoglobin. The black solid line represents the OR, while the gray shaded area indicates the 95% confidence interval (CI). The red dotted line denotes the reference point where OR = 1.
(PDF)
