# Supplementary figures and images for: Assessing the impact of sodium intake on kidney function deterioration and proteinuria in the general population: A prospective cohort study
Source: PLoS One. 2025 Aug 29;20(8):e0330342. doi: 10.1371/journal.pone.0330342 (PMC12396720; doi:10.1371/journal.pone.0330342)

**A**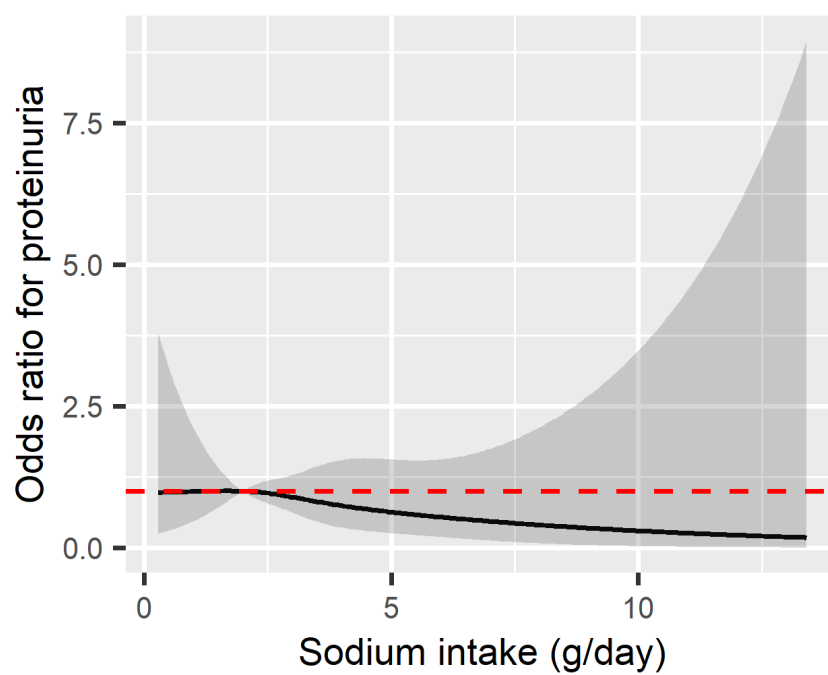**B**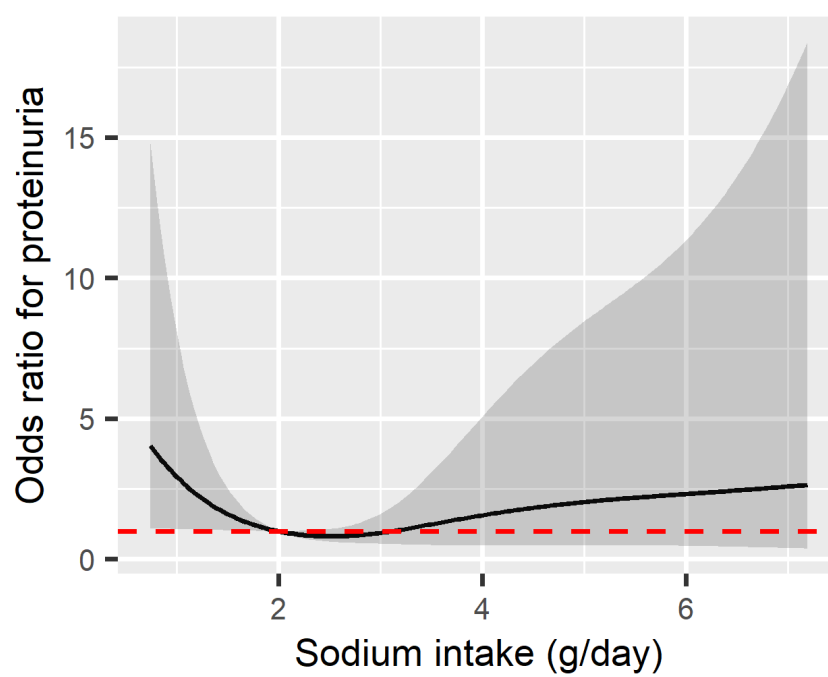

Supplement: S1 Fig — S1A and S1B Figs show individuals without and with diabetes mellitus, respectively. Odds ratio (ORs) are adjusted for age, sex, body mass index (BMI), smoking status, alcohol consumption, hypertension, diabetes, coronary artery disease, pre-existing kidney disease, baseline estimated glomerular filtration rate (eGFR), blood albumin level, and hemoglobin. The black solid line represents the OR, while the gray shaded area indicates the 95% confidence interval (CI). The red dotted line denotes the reference point where OR = 1. (PDF) [file pone.0330342.s001.pdf]

**A**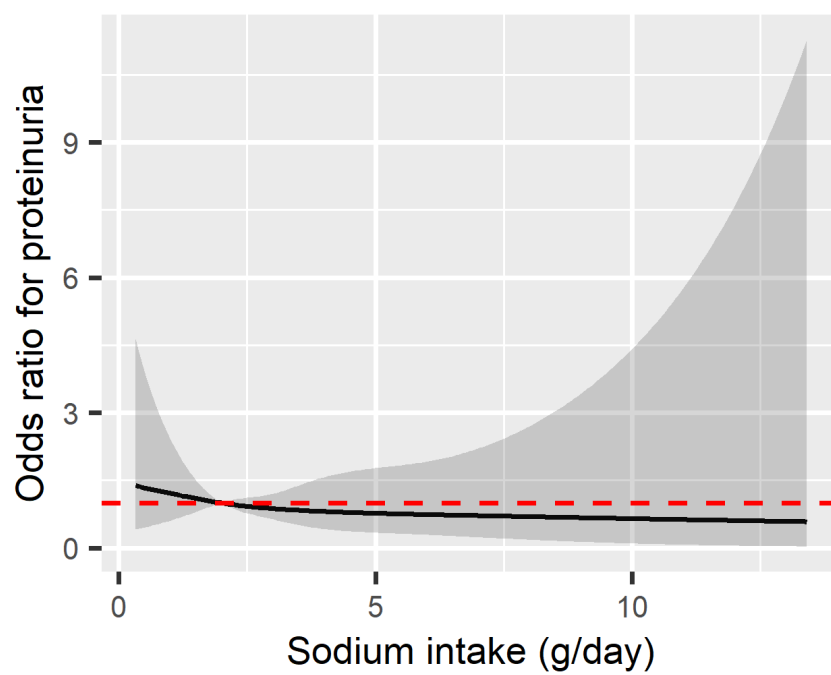**B**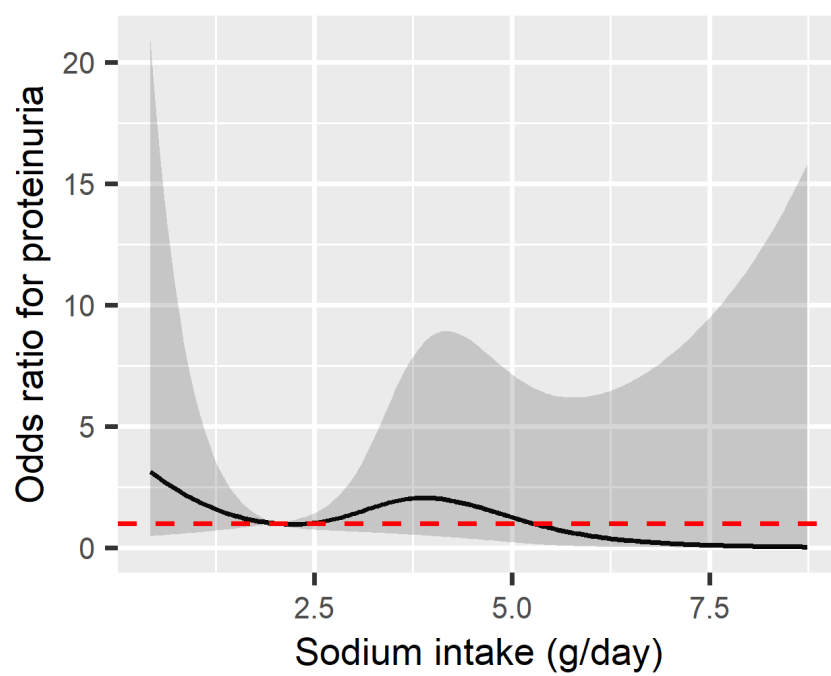

Supplement: S2 Fig — S2A and S2B figures show individuals without and with hypertension, respectively. Odds ratio (ORs) are adjusted for age, sex, body mass index (BMI), smoking status, alcohol consumption, hypertension, diabetes, coronary artery disease, pre-existing kidney disease, baseline estimated glomerular filtration rate (eGFR), blood albumin level, and hemoglobin. The black solid line represents the OR, while the gray shaded area indicates the 95% confidence interval (CI). The red dotted line denotes the reference point where OR = 1. (PDF) [file pone.0330342.s002.pdf]
